# Attentional Capture to a Singleton Distractor Degrades Visual Marking in Visual Search

**DOI:** 10.3389/fpsyg.2017.00801

**Published:** 2017-05-16

**Authors:** Kenji Yamauchi, Takayuki Osugi, Ikuya Murakami

**Affiliations:** ^1^Department of Psychology, The University of TokyoTokyo, Japan; ^2^Japan Society for the Promotion of ScienceTokyo, Japan

**Keywords:** visual marking, singleton, attentional capture, visual search, preview benefit

## Abstract

Visual search is easier after observing some distractors in advance; it is as if the previewed distractors were excluded from the search. This effect is referred to as the preview benefit, and a memory template that visually marks the old locations of the distractors is thought to help in prioritizing the locations of newly presented items. One remaining question is whether the presence of a conspicuous item during the sequential shift of attention within the new items reduces this preview benefit. To address this issue, we combined the above preview search and a conventional visual search paradigm using a singleton distractor and examined whether the search performance was affected by the presence of the singleton. The results showed that the slope of reaction time as a function of set size became steeper in the presence of a singleton, indicating that the singleton distractor reduced the preview benefit. Furthermore, this degradation effect was positively correlated with the degree of conventional attentional capture to a singleton measured in a separate experiment with simultaneous search. These findings suggest that the mechanism of visual marking shares common attentional resources with the search process.

## Introduction

Because the visual system has a limited capacity, attention is often used to restrict the range of search for a target within a subset of regions or objects. Such selective attention has been psychophysically investigated by using a visual search task. In this task, observers are required to search for a specific target among distractors. During search, attention tends to be automatically drawn to salient objects, such as those that appear abruptly (Yantis and Jonides, 1984) and those that differ from other items (Theeuwes, 1992, 2004). This tendency is called *attentional capture*.

On the other hand, attention can also be directed with top-down control based on the observer’s knowledge about pertinent stimulus features of a target and his will to restrict the search behavior within only a subset of stimuli sharing the same features as the target (Egeth et al., 1984; Kaptein et al., 1995; Sobel and Cave, 2002). The visual system can also use positional memory instructing where not to search for the target, to optimize the strategy of prioritizing selection for a subset of objects. This top-down strategy is called *visual marking*. Watson and Humphreys (1997) demonstrated this function using a preview search task. In this task, a subset of distractors (“old items”) appears in the initial display frame followed by the additional onset of the remaining distractors and the target (“new items”) at locations that have not been occupied by the old items. Search performance in this condition, measured by reaction times (RTs) as a function of the total number of items (“set size”), is significantly better than when all items appear simultaneously. This beneficial phenomenon is referred to as the *preview benefit* (Watson et al., 2003). Furthermore, Theeuwes et al. (1998) manipulated the numbers of new and old items, demonstrating that the RTs depended on the number of new items but not on the number of old items; it was as if the old items were all ignored and excluded from the search.

Watson and Humphreys (1997) argued that preview benefit reflects some active inhibitory bias applied to the locations of old items. However, several studies suggested that the preview benefit can occur due either to automatic attentional capture triggered by the onset of new items (e.g., Donk and Theeuwes, 2001, 2003; Atchley et al., 2003) or due to the mere temporal grouping of new items resulting in the perceptual segmentation of new and old items during asynchronous presentations, without active inhibition of old items (e.g., Jiang et al., 2002). Although there is evidence for some contribution of both onset and grouping to the preview benefit, these accounts alone cannot explain the following findings from previous studies. First, when observers are required to perform a secondary task to detect a dot stimulus added to the search display, a dot placed near the location of a new item is more readily detectable than that placed near the location of an old item is (Watson and Humphreys, 2000; Allen and Humphreys, 2007; Osugi et al., 2009; Osugi and Murakami, 2014). Second, preview benefit, as determined by RT reduction for the target presented as a new item, is more effective when the observer is informed that the target will always appear at one of the locations of new items than when she is informed that the target can appear either at one of the new-item locations or at one of the old-item locations at equal likelihoods (Osugi et al., 2016). Third, color-based inhibition is unintentionally carried over to new items in the search display if they are in the same color as old items in the preview display (Braithwaite and Humphreys, 2003; Braithwaite et al., 2005). These studies suggest that inhibitory visual marking normally works in concert with attentional capture and plays a substantial role in prioritizing the selection of new items (Watson et al., 2003).

Visual marking has three important characteristics. First, it requires attentional resources during the preview period to set up and maintain an internal map of the locations of old items that are ignored later. For example, the preview benefit is degraded when observers have to perform a secondary task during the preview period, suggesting that visual marking involves an active attentional process to mark the locations of the old items while they are being previewed (Watson and Humphreys, 1997; Humphreys et al., 2002; Olivers and Humphreys, 2002). Second, visual marking is automatically disrupted if attention is captured by changes in the old items (Watson and Humphreys, 1997, 2002) or changes in a background region (Osugi and Murakami, 2015) that occur simultaneously with the onset of new items. This happens even when the luminance of the old items is maintained as their shapes are abruptly changed. However, the disruption of visual marking by such changes is less severe if they involve either eye blink (Irwin and Humphreys, 2013; von Mühlenen et al., 2013), occlusion (Kunar et al., 2003), or transient masking (Watson and Kunar, 2010). Third, visual marking is retained for a fairly long time. For example, the maximum preview benefit is observed even in very large set sizes requiring long inspection time (Watson and Humphreys, 1997; Theeuwes et al., 1998). Additionally, eye fixations are biased against old items during each search trial lasting 5 s, when old and new items differ in color (Watson and Inglis, 2007), and the first four saccades tend to avoid old items when all items are of the same color (Emrich et al., 2008).

Watson et al. (2003) proposed that visual marking is established by a top-down goal-based inhibitory process. This framework assumes that the inhibitory template, in which the locations of old items are encoded and maintained, plays a role in ignoring old items during the subsequent search process, and that the encoding and maintenance of the inhibitory template requires attentional resources. However, this model does not explain how to maintain the inhibitory template throughout the search process.

The central question here is how the attentional resources spent for the maintenance of visual marking and those used throughout the search process relate to each other. One hypothesis is that the attentional resources for search are independent of those for visual marking; this view posits that the search task does not interfere with the maintenance of an inhibitory template, which explains why visual marking is retained for a fairly long time. An alternative hypothesis is that the search task and visual marking compete for common attentional resources and that visual marking is impaired when excessive attentional resources are consumed for the search. To test these hypotheses, it is necessary to use a paradigm in which attentional resources must be largely consumed for the search task, such as search for a target among distractors containing a color singleton that captures attention (Theeuwes, 1991, 1992). In an exemplar situation, observers are required to search for a target (e.g., a green diamond) among distractors (e.g., green circles). In each singleton trial, one of the distractors markedly differs from the others in appearance (e.g., a red circle)—hence, a singleton—whereas no singleton is presented in the remaining trials. RTs become longer in the presence of a task-irrelevant singleton, indicating that such a singleton serves as an exogenous cue to capture spatial attention and interferes with search. Thus, we found this a useful situation to assess whether a transient perturbation of attentional resources in the search process degrades the preview benefit, and we combined the preview search task with an additional singleton paradigm in which a task-irrelevant color singleton appeared in the search display.

As such, we tested whether attentional capture to a singleton distractor steals some attentional resources for visual marking and degrades it in the “preview search,” in which half of the items appeared in advance in the preview display. These were followed by the search display in which the remaining items were added to the old items. We used a serial search task because the preview benefit had to be assessed from the set size dependence of RT. Attentional capture to a task-irrelevant color singleton robustly occurs in parallel search (Theeuwes, 1991, 1992), but its occurrence in serial search is controversial; some studies found a vigorous RT cost (e.g., Theeuwes and Burger, 1998), while others did not show much of a cost (e.g., Bacon and Egeth, 1994; Yantis and Egeth, 1999). However, researchers have argued that the lack of overt attentional capture does not necessarily mean that a color singleton does not affect attentional resources. For example, event-related potential studies have suggested that a singleton is actively suppressed in serial search by showing that an inhibition-related component, called distractor positivity, is larger in trials in which behavioral RT is shorter (Sawaki and Luck, 2010, 2014; Gaspar and McDonald, 2014). Furthermore, when perceptual suppression was assessed by a probe detection technique, reported probe letters were less accurate when they were presented at a singleton’s location than when they were presented at a non-singleton distractor’s location, even when RT cost itself was absent in the serial search (Gaspelin et al., 2015). These findings suggest that, even if an overt performance change is absent, a color singleton can covertly steal attentional resources during serial search, but they are so quickly “withdrawn” or “actively suppressed” by the top-down control that no performance change is detected experimentally (Theeuwes and Burger, 1998; Barras and Kerzel, 2016). That said, we were interested to compare performances on preview search with those on “simultaneous search,” in which all items appeared at once and attentional capture to a color singleton distractor was expected to occur. Because RT cost in the presence of a singleton distractor can exhibit fairly large individual differences (e.g., Fukuda and Vogel, 2009), interobserver correlations might be able to reveal a relationship between RT cost as an index of attentional capture in the simultaneous search task and the preview benefit in the preview search task. Therefore, this study consisted of two experiments. In Experiment 1, we used a preview search task in which the new items could contain a color singleton distractor, and examined whether it affected the preview benefit. In Experiment 2, we used a simultaneous search task in which items could contain a color singleton, and determined the occurrence of attentional capture with RT cost as an index.

## Materials and Methods

### Observers

Eighteen adults (aged 19–32 years) who were naïve to the purpose of the study participated, in addition to the first and second authors. All observers had normal or corrected-to-normal visual acuity and color vision, and they were familiar with the letters of the English alphabet. This study was carried out in accordance with the recommendations of the Ethical Principles of American Psychological Association and the Declaration of Helsinki, and written informed consent was secured from all participants. The protocol was approved by the institutional ethics committee of the Graduate School of Humanities and Sociology at the University of Tokyo.

### Stimuli and Apparatus

Stimuli were displayed on a CRT monitor (Mitsubishi Electric RDF223H, 1024 × 768 pixels) controlled by a computer using a MATLAB^®^ programming environment (MathWorks) and the Psychophysics Toolbox extensions (Brainard, 1997; Pelli, 1997; Kleiner et al., 2007). The refresh rate of the monitor was 60 Hz. The viewing distance was 57 cm. A white (81.5 cd/m^2^) fixation dot (0.23° × 0.23°) was presented at the center of the display. The search items were red (20.36 cd/m^2^, *x* = 0.627, *y* = 0.341) or green (20.36 cd/m^2^, *x* = 0.281, *y* = 0.611) uppercase letters, each subtending 2° in height and 1° in width, with each line segment 0.16° wide, and they were presented on a black background (<0.01 cd/m^2^). The target was either an “H” or a “U,” and the distractors were “C,” “E,” “A,” “P,” “O,” “F,” and “S.” The items were presented at pseudo-randomly selected locations out of 48 possible locations (except for the fixation-dot location) in an invisible 7 × 7 matrix subtending 21.35° in height and width. In each trial, random spatial offsets were added to stimulus positions within a range of ±0.16° horizontally and ±0.08° vertically. The target could appear at any of these locations with equal likelihood.

### Behavioral Task

Before the experiment began, the observers were informed of the details of the search conditions included in each experimental block. They were asked to search for “H” or “U” and to identify its shape as quickly as possible by pressing the “Z” or “M” key on a computer keyboard to indicate “H” or “U,” respectively. RTs were measured. When the response was incorrect or when the RT was longer than 5,000 ms, a 1,000-Hz tone was presented for 20 ms to indicate the abortion of the trial.

### Experiment 1: Preview Search Task

In Experiment 1, we examined whether the search performance in the preview search task was altered by a color singleton distractor presented within the new items. We measured the RTs required to search for the target and used the slope of RT as a function of set size to determine the preview benefit (Theeuwes et al., 1998).

**FIGURE 1:**
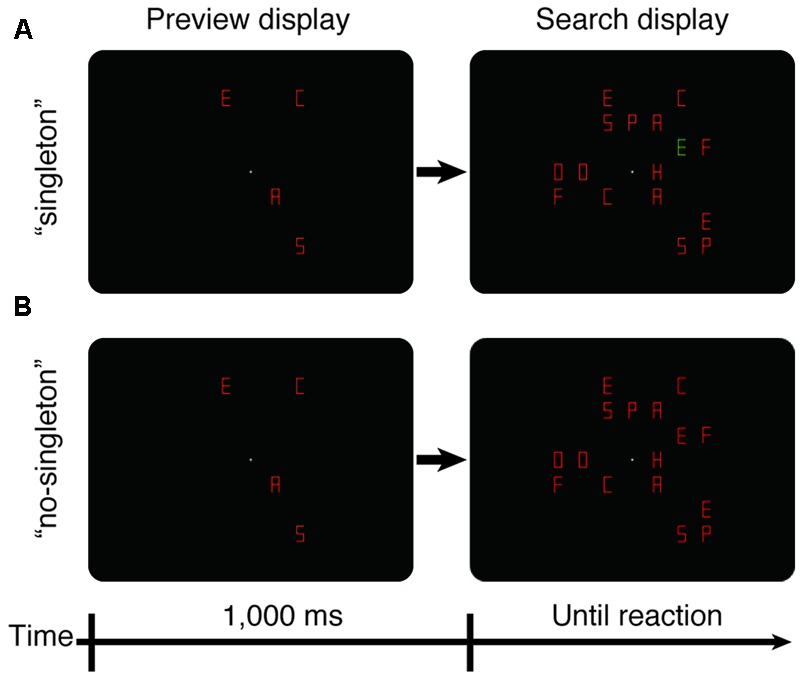
**Schematics of stimulus displays for the preview search (Experiment 1). (A)** The “singleton” condition. **(B)** The “no-singleton” condition. First, the preview display was presented for 1,000 ms. The preview display contained only distractors. Next, the search display was presented and remained until either the observer’s reaction or the time limit of 5,000 ms. The search display contained the target and the remaining distractors. In the simultaneous search (Experiment 2), the stimulus displays were identical, except that the preview display was not presented.

Experiment 1 involved a within-observer 2 × 3 design: two singleton conditions (“singleton” and “no-singleton”) and three set sizes (16, 20, and 24). Along with set size, the number of the old items was varied (it could be 4, 8, or 12), while the number of new items was fixed at 12. Each trial began with the presentation of a fixation dot for 500 ms, followed by the onset of the old items (**Figure 1**). After a stimulus-onset asynchrony of 1,000 ms, new items, including the target, were added at previously blank locations. In the “singleton” condition, the old items and 11 new items, including the target, were in one color, and the remaining one within the new items was in the other color—hence, a color singleton. Because alternating colors maximize interference from the color singleton (Kerzel and Barras, 2015), the singleton was always a distractor and was either red among green or green among red with equal likelihood. In the “no-singleton” condition, all items were in the same color (either red or green). Each experimental block consisted of a total of 48 trials (2 singleton conditions × 3 set sizes × 2 target letters × 2 target colors × 2 repeated trials) presented in a random order. Each observer completed eight such blocks within 1 day. The first two blocks were spent as practice sessions, and the data of the succeeding six blocks were used for the analysis.

If the preview benefit occurred maximally, the old items would be completely ignored, and thus, the RT slope would be flat, irrespective of set size. If preview benefit occurred despite attentional capture to the singleton, the RT slope would be the same under both conditions. If attentional capture to the singleton stole some portion of attentional resources that had been used to maintain the inhibitory template for visual marking, the search slope under the “singleton” condition would become steeper.

### Experiment 2: Simultaneous Search Task

In Experiment 2, we examined whether the search performance in the simultaneous search task would be altered by a color singleton distractor. All observers who participated in Experiment 1 also participated in Experiment 2, after an interval of 13–53 days. If attentional capture to the singleton occurred overtly, the RT would be longer with the singleton than without it. If the singleton did not have a sufficiently strong impact to cause overt attentional capture, the RTs under the two conditions would be the same.

Experiment 2 involved a within-observer 2 × 3 design: two singleton conditions (“singleton” and “no-singleton”) and three set sizes (16, 20, and 24). Each trial began with the presentation of the fixation dot for 1,500 ms, followed by the simultaneous onset of all items. In the “singleton” condition, one of the distractors was a color singleton that differed in color (e.g., red) from the other items (e.g., green). In the “no-singleton” condition, all items were of the same color. Each experimental block consisted of a total of 48 trials (2 singleton conditions × 3 set sizes × 2 target letters × 2 target colors × 2 repeated trials), presented in a random order. Each observer completed eight such blocks within 1 day. The first two blocks were spent as practice sessions, and the data of the succeeding six blocks were used for the analysis.

## Results

The RTs for incorrect responses and the trials in which no reaction occurred within 5,000 ms (<0.3% of all trials) were considered erroneous and were excluded from the analysis. The error data generally followed the same trends as the RT data and did not suggest any speed–accuracy trade-off; thus, no further analysis of the errors has been provided.

### Experiment 1: Preview Search Task

**FIGURE 2:**
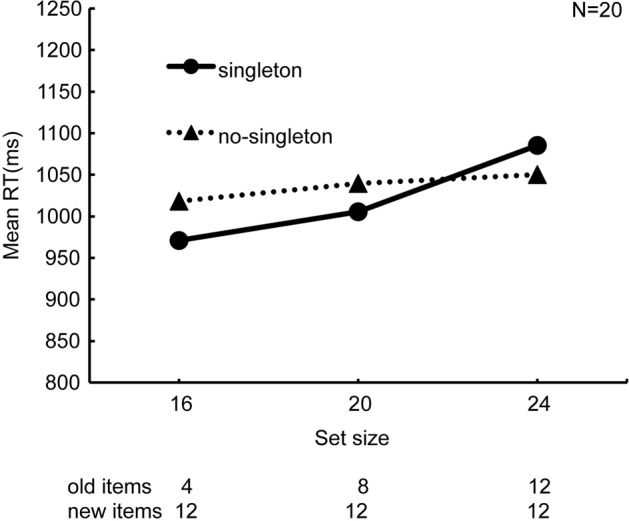
**Results of Experiment 1, namely the preview search, for all the observers (*N* = 20).** Set size, i.e., the total number of items, and the respective number of the old items are described along the abscissa. Note that the number of the new items was fixed at 12. The equation of the regression line in the “singleton” condition is *y* = 14.62x + 906.16, and that in the “no-singleton” condition is *y* = 3.99x + 1003.88.

In **Figure 2**, RTs averaged across all the observers are plotted against set size. The search slope, determined by a linear regression of RT as a function of set size, was steeper in the “singleton” condition (14.62 ms/item) than in the “no-singleton” condition (3.99 ms/item). A 2 × 3 ANOVA with singleton (“singleton” and “no-singleton”) and set size (16, 20, and 24) as within-observer factors revealed that the main effect of set size (*F*_2,38_ = 5.99, *p* < 0.01, $\eta_{p}^{2}$ = 0.24) and the interaction (*F*_2,38_ = 3.61, *p* < 0.05, $\eta_{p}^{2}$ = 0.16) were significant, whereas the main effect of singleton was not (*F*_1,19_ = 0.40, *p* = 0.53, $\eta_{p}^{2}$ = 0.01). The simple main effect of set size was significant in the “singleton” condition (*F*_2,76_ = 9.45, *p* < 0.01, $\eta_{p}^{2}$ = 0.20), but not in the “no-singleton” condition (*F*_2,76_ = 0.71, *p* = 0.50, $\eta_{p}^{2}$ = 0.02). Because the maximal preview benefit by visual marking predicts a flat slope, these results indicate that the preview benefit maximally occurred under the “no-singleton” condition, and that it was compromised under the “singleton” condition.

### Experiment 2: Simultaneous Search Task

**FIGURE 3:**
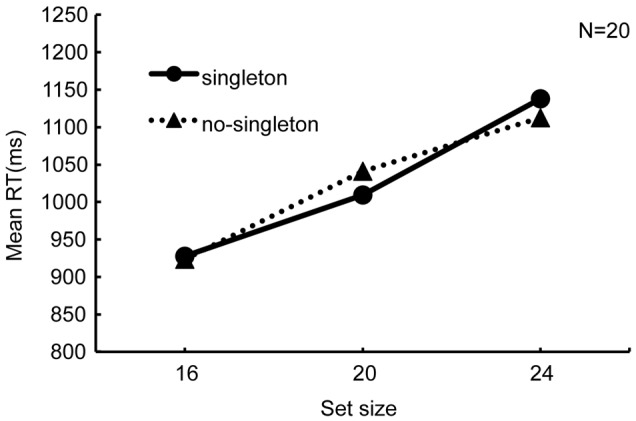
**Results of Experiment 2, namely the simultaneous search, for all the observers (*N* = 20).** Reaction time is plotted against set size with the singleton factor as a parameter. The equation of the regression line in the “singleton” condition is *y* = 26.61x + 814.74, and that in the “no-singleton” condition is *y* = 23.60x + 836.87.

**Figure 3** shows RTs against set size. If the singleton distractor captured attention, the RTs would increase under the “singleton” condition. However, the two search functions appeared similar to each other. The search slope in the “singleton” condition was 26.61 ms/item and that in the “no-singleton” condition was 23.60 ms/item. A 2 × 3 analysis of variance (ANOVA) with singleton (“singleton” and “no-singleton”) and set size (16, 20, and 24) as within-observer factors revealed a significant main effect of set size (*F*_2,38_ = 49.33, *p* < 0.01, $\eta_{p}^{2}$ = 0.72) but neither a main effect of singleton (*F*_1,19_ = 0.01, *p* = 0.92, $\eta_{p}^{2}$ = 0.0003) nor their interaction (*F*_2,38_ = 1.37, *p* = 0.27, $\eta_{p}^{2}$ = 0.07). Thus, the group analysis failed to show that the singleton distractor affected search performance.

**FIGURE 4:**
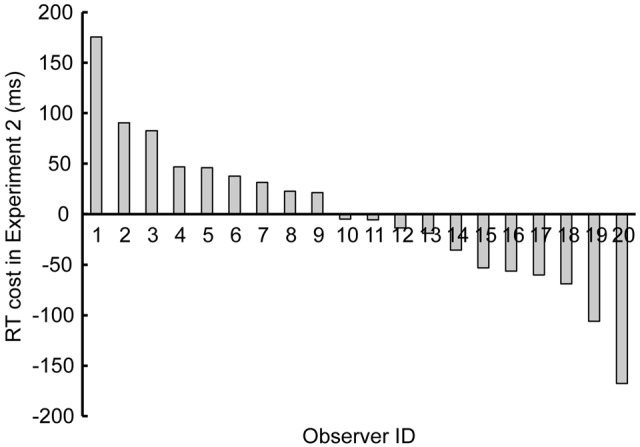
**Results of Experiment 2 showing individual variability in reaction time (RT) cost, i.e., the RT in the “singleton” condition minus the RT in the “no-singleton” condition, both averaged across set size.** Each bar corresponds to each observer, sorted in the descending order.

**Figure 4** shows the individual variability in “RT cost” by the singleton, i.e., the RT in the “singleton” condition minus the RT in the “no-singleton” condition, both averaged across set size. Nine observers (#1–9) exhibited nominally positive RT costs, whereas the other 11 observers (#10–20) exhibited nominally negative RT costs.

**FIGURE 5:**
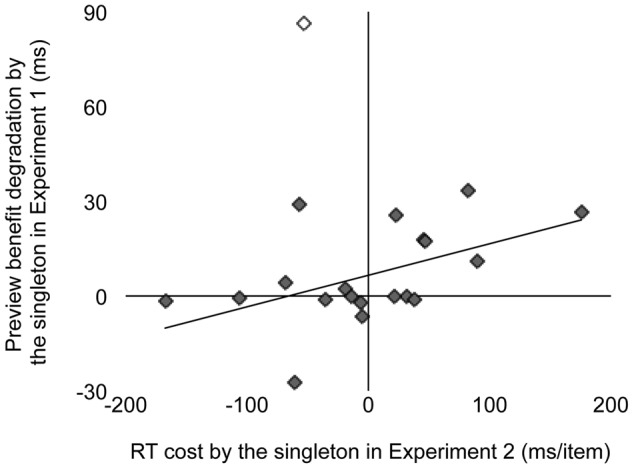
**Interobserver scattergram showing a correlation between reaction time (RT) cost by the singleton (Experiment 2) and preview benefit degradation by the singleton (Experiment 1).** RT cost was replotted from **Figure 4**. Preview benefit degradation was defined as the slope in the “singleton” condition minus the slope in the “no-singleton” condition. The open symbol indicates the observer we excluded from the correlation analysis as an outlier. The line shows the best-fit linear regression.

We examined whether the individual variability in RT cost in Experiment 2 predicted the individual variability in the preview benefit degradation in Experiment 1. Degradation was quantified by the slope in the “singleton” condition minus the slope in the “no-singleton” condition in Experiment 1. **Figure 5** shows a scattergram between RT cost and preview benefit degradation. Because inspection by eye detected an outlier who might have affected the correlation, we removed this observer (out of ±3 SD around mean; open symbol) and calculated the correlation. There was a significant positive correlation between RT cost and preview benefit degradation (*r* = 0.51, *p* < 0.03), meaning that the individual differences had information of their own, in addition to the effect of random noise that was predicted from the study by Anderson and Folk (2010), who suggested that there are substantial trial-to-trial differences in the degree of attentional capture. Therefore, the correlation was consistent with the interpretation that attentional capture to a singleton distractor degraded preview benefit.

## Discussion

This study examined whether the competition of attentional resources between attentional capture and visual marking degrades the preview benefit due to the latter. In Experiment 1, to test whether attentional capture to a singleton degraded visual marking, we analyzed the results in the preview search task in which the presentation of some distractors was followed by the onset of the other distractors and the target, with or without an additional singleton distractor. We successfully replicated the conventional visual marking because RT did not depend on the number of old distractors previewed before the search was initiated. However, RT increased with set size when the search display contained a singleton distractor, indicating compromise of the preview benefit (**Figure 2**). In Experiment 2, when all stimuli appeared simultaneously, the group analysis failed to replicate the conventional attentional capture to a singleton distractor because RT did not significantly change with or without the singleton (**Figure 3**). However, there was also noticeable individual variability in RT cost (**Figure 4**). Therefore, there were large individual differences pertaining to whether the singleton caused overt attentional capture, consistent with Fukuda and Vogel’s (2009) study indicating that the ability to override attentional capture varies widely from person to person. There was a significant positive correlation between RT cost and preview benefit degradation, suggesting that attentional capture to a singleton distractor degrades preview benefit (**Figure 5**). These findings are consistent with the hypothesis that attentional capture to a color singleton degrades the attentional resources necessary to maintain the inhibitory template for visual marking.

According to Watson and Humphreys (1997), attentional resources are required to set up and maintain the inhibitory template. Thus, the template could be removed when attentional resources are exhausted by being engaged in a secondary task (e.g., Olivers and Humphreys, 2002). However, previous studies have only examined the effects of a secondary task engagement during the preview period prior to the appearance of the new items, not during the subsequent search period. The present study demonstrates that the inhibitory template can be degraded by attentional capture to a singleton distractor during the search. Though the visual system forms an inhibitory template when old items are presented for a long time (Watson and Humphreys, 1997), attentional resources to maintain the inhibitory template can be exhausted when the system has to deal with the additional presentation of new items, including a singleton capturing attention. However, such reallocation of attentional resources to a singleton may be overridden by top-down control that actively suppresses the singleton distractor immediately after its detection (Belopolsky et al., 2010; Gaspar and McDonald, 2014; Sawaki and Luck, 2010, 2014; Gaspelin et al., 2015; Barras and Kerzel, 2016). We speculate that those observers who exhibited shorter RTs with a singleton than without it may have been equipped with better top-down control capabilities. Then, our correlation analysis would imply that during the search process, quick and active suppression of a singleton by top-down control may spend certain attentional resources but do not necessarily compete for the maintenance of an inhibitory template for visual marking, judging from the tendency that those observers with shorter RTs with a singleton in the simultaneous search did not systematically show compromise of preview benefit in the preview search (i.e., **Figure 4** did not show a V-shaped correlation structure that should have emerged if both positive and negative RT costs had led to resource competition with visual marking).

A previous study measured performance for selecting and responding to all the new stimuli rather than a single target item within the new set (Watson and Kunar, 2012); even when actions were allowed 3 s after the onset of the new items, correct selection responses for the new items were reduced only by approximately one item, suggesting that time alone can cause only a slight degradation of the representation for visual marking, consistent with a known characteristic of visual marking that it is retained for a fairly long time (Watson and Humphreys, 1997; Theeuwes et al., 1998). In contrast, localizing and responding to new items and executing saccades can somewhat reduce the preview benefit (Emrich et al., 2008; Watson and Kunar, 2012), suggesting that multiple responses to new items and/or sequential shifts of attention interfere with the representation for visual marking. Likewise, the present study demonstrates that the singleton distractor among new items draws attention and interferes with visual marking functionality. These findings suggest that not only time but also attention-demanding processes cause degradation of preview benefit.

Previous studies have demonstrated that the preview benefit is degraded when the shapes of old items are changed at the onset of new items (Watson and Humphreys, 1997, 2002) or when the background is changed from static random noise to dynamic random noise (Osugi and Murakami, 2015), suggesting that attentional capture to an abrupt event in the scene is a crucial factor to control the efficiency of the preview benefit. However, it remained unclear whether attentional capture is really involved or whether stimulus change itself is sufficient. The present findings clarified this concern by demonstrating that attentional capture to a color singleton degraded the preview benefit. In our experiment, there were no stimulus changes between the preview and search displays, except for the addition of new items, which was obviously an inevitable constituent of the preview search paradigm and was controlled across conditions. On the other hand, several studies demonstrated that the preview benefit survives stimulus changes if they involve eye blink (Irwin and Humphreys, 2013; von Mühlenen et al., 2013), occlusion (Kunar et al., 2003), or transient masking (Watson and Kunar, 2010). Taken together, not only bottom-up signals about stimulus changes but also higher-order generative processes for visual scene construction and maintenance are likely involved in attentional capture disturbing visual marking. Our visual system is designed to make the maximal use of limited attentional capacity by reallocating attentional resources to scrutinize a potentially interesting/threatening event in the outer world, e.g., a stimulus change at marked object locations, a change in dynamics in the background, and a singleton popping out among new objects, at the expense of search efficiency gained by sticking to the memory template for visual marking.

What underlying mechanisms might support these resource allocation functions? Watson and Humphreys (1997) proposed that visual marking is achieved by top-down control that biases one’s attentional set toward a subset of objects and is removed by subsequent bottom-up activities in the visual system, which reports novel events such as sudden onsets or offsets of stimuli. We would argue that there are at least two processing routes to make this marking removal possible. One route is a retino/spatiotopic link between the top-down memory template and bottom-up visual signals, as was assumed in the theory by Watson and Humphreys (1997), sharing a common reference map of the visual world, perhaps a salience or priority map (e.g., Fecteau and Munoz, 2006). Thus, attentional capture to a dynamic event occurring therein facilitates the mechanism for visual marking to purposefully remove inhibitory marking upon modification of the values in the map at the locations of old items. Another route involves a supervisory resource allocation mechanism among multiple top-down control systems and does not refer to location-specific modifications. Upon request from attentional processing that deals with a singleton distractor, a certain proportion of attentional resources already allocated to visual marking is forcibly recycled for this novel use, and thus the maintenance of inhibitory marking becomes more difficult. In future studies, the involvement of these two routes may be clarified through functional brain imaging, which will enable distinction between retino/spatiotopic visual maps and cognitive conflict control systems in terms of the differences in the activated cortical areas (e.g., Tootell et al., 1998; Fan et al., 2005).

In the preview search task, the intercept of the search function was lower with the singleton than without it. Although a similar effect has been noted when observers are strongly motivated to ignore a singleton distractor (Gaspelin et al., 2015), the reason why this phenomenon occurs remains unclear. Nonetheless, we would speculate two possible reasons. First, this benefit might have occurred because attentional resources were quickly—much more quickly than the time needed for the serial search of each item—reallocated from the singleton to the remaining items. In this case, observers could have searched for the target among N - 1 items throughout all set sizes, and thus RT would have been constantly shortened in the singleton present trials. Second, the presence of a singleton might have been judged more quickly than the absence of it. If this were the case, participants could have commenced search earlier under the singleton condition, resulting in a decrease in intercept.

## Conclusion

This study revealed that attentional capture to a singleton during search is one of the factors that can degrade visual marking. The memory template for visual marking may have a large capacity and a long retention time, but it can be disrupted by a single conspicuous visual object encountered during search. This suggests the vulnerability of visual marking in a cluttered scene in everyday life with a lot of natural as well as artificial variability in feature dimensions among visual objects. Nevertheless, considering the lack of overtraining in the observers of our study, there is a possibility for a beneficial effect of long-term learning of preview search to overcome such a limitation.

## Author Contributions

KY conducted the experiment, analyzed the data, and wrote the manuscript. TO planned the experimental design, developed the computer programs for the experiment, and wrote the manuscript. IM supervised the research, contributed to the interpretation of the data, and wrote the manuscript.

## Conflict of Interest Statement

The authors declare that the research was conducted in the absence of any commercial or financial relationships that could be construed as a potential conflict of interest.
